# Diabetic Microvascular Complications Among Children and Adolescents in Northwestern Tanzania: A Cross-Sectional Study

**DOI:** 10.5334/aogh.2669

**Published:** 2020-04-24

**Authors:** Delfina Msanga, Karl Reis, Neema Kayange, Respicious Bakalemwa, Benson Kidenya, Duncan Hau, Christopher Mwanansao, Dina Mahamba, Sofia Ottaru, Elizabeth Kwiyolecha, Robert Peck

**Affiliations:** 1 Weill Bugando School of Medicine, Mwanza, TZ; 2 Center for Global Health, Division of Medicine, Weill Cornell Medical College, New York, NY, US; 3 University of Dodoma, Dodoma, TZ

## Abstract

**Background:**

Africa is experiencing a rapid increase in morbidity and mortality related to diabetes mellitus (DM). Contemporary data are needed to guide efforts to improve prevention and treatment for microvascular complications in children and adolescents in Africa. This study was conducted to assess prevalence of diabetic microvascular complications in northwestern Tanzania, including nephropathy, retinopathy, and neuropathy, as well as associated risk factors.

**Objectives:**

1) To determine the prevalence of microvascular complications and the overlap of nephropathy, retinopathy and neuropathy and 2) to determine factors associated with the development of microvascular complications.

**Methods:**

This cross-sectional study included 155 children and adolescents with DM consecutively attending all three health centers providing diabetes care for children in the Mwanza region of Tanzania. Participants were examined for microvascular complications and possible risk factors.

**Results:**

Fifty-one of 155 participants (age: 5–19 years) had diabetic nephropathy (32.9%), 16 had diabetic retinopathy (10.3%), and 21 had diabetic neuropathy (13.6%). Risk factors for development of a microvascular complication included age, duration of DM, and poor glycemic control. Of the participants, 107 had poor levels of glycemic control (69%) with HbA1C levels >10%.

**Conclusion:**

The prevalence of microvascular complications, especially that of nephropathy, was disturbingly high. Risk factors for microvascular complications were similar to other studies from Africa and included poor glycemic control, older age, and longer duration of DM. Innovative, locally appropriate systems for optimizing glycemic control are urgently needed.

## Background

Africa is experiencing a rapid increase in morbidity and mortality related to diabetes mellitus (DM) [1]. The number of individuals living with DM in Sub-Saharan Africa (SSA) is expected to grow from 12.1 million in 2010 to 34.2 million by 2040, the largest percentage increase in DM of any region in the world [2]. DM is particularly problematic in this part of the world due to the fragility of the health systems [3] and the many associations between DM and infectious diseases prevalent in SSA [1,4,5].

Outcomes for children with DM in Africa are poor [6]. Death from diabetic ketoacidosis or hypoglycemia is common, as are long-term complications like cognitive dysfunction, growth stunting, pubertal delay, and infectious complications [7,8]. The life expectancies of children with diabetes are also significantly reduced [2]. One study estimated that children in rural Mozambique with DM live on average just 7.2 months after diagnosis [9].

Despite the fact that microvascular complications are an important source of DM related morbidity and mortality in children in Africa, little is known about the prevalence of these complications [10]. The results of the studies that have been done tend to differ significantly, suggesting the need for further research [3]. Children in Africa are already at significant risks for conditions such as kidney disease, where the average prevalence of proteinuria in children is 32.5%, higher than in adults in the USA and SSA [11]. This background rate of disease makes DM-related complications an especially important source of risk. Moreover, microvascular complications are a significant economic burden on both individuals and health systems as treating them is more expensive then treating controlled DM and demands a higher level of expertise [3].

Therefore, we conducted a cross sectional study of children and adolescents with DM to increase understanding of microvascular complications in Tanzania. Our objectives were: 1) to determine the prevalence of microvascular complications and the overlap of nephropathy, retinopathy, and neuropathy and 2) to determine factors associated with the development of microvascular complications.

## Methods

### Study area

This research was conducted in the outpatient clinics of three health facilities in Mwanza, Tanzania: 1) Bugando Medical Center, a tertiary hospital serving the Lake Zone Region; 2) Sekou Toure Hospital, a regional referral hospital serving the seven districts of Mwanza; and 3) Sengerema Hospital, a district hospital serving the district of Sengerema.

### Study population

The study population consisted of children and adolescents ages 1–19 years with DM attending the three specified outpatient clinics. Exclusion criteria were less than one year of age, a urinalysis indicating possible urinary tract infection, or a temperature of 38 degrees or above.

### Study procedures

All participants enrolled in the study were interviewed using a pre-tested questionnaire. Demographic information, including age, sex, residence location (classified as rural or urban, depending on proximity to the local urban center), and family history of diabetes mellitus were collected. Next, a medical history was obtained from every participant, including time since DM diagnosis, the dose/dose frequency of insulin, and insulin storage habits. We then asked whether the patient had a history of painful/hot sensations on either the lower or upper extremities. Lastly, results of any previous renal or eye exams were recorded.

Anthropometric measurements including height, weight, and vital signs were performed on each participant. Height of children aged 2–19 and length of children aged 1–2 was measured with a ShorrBoard measuring board (Weight and Measure, LLC, Maryland, USA). Weight was measured using a DETECTO scale (Web City, USA), which was calibrated before each use. Using this information, BMIs were calculated and interpreted according to the age specific WHO chart [12]. Blood pressure was measured using a manual sphygmomanometer with an appropriately sized cuff in sitting and standing positions using standard technique [13]. To check for peripheral neuropathy, a Semmes Weinstein test was performed with a 10g monofilament. The filament was first shown to the participant and used on the wrist to ensure they were comfortable with the procedure. The participant was instructed to notify the test-administrator when they perceived the pressure of the filament. The filament was then applied perpendicularly to the skin on four different areas of the sole of each foot. Three or less correct responses was considered to be indicative of neuropathy. Lastly, a digital fundoscopy exam was performed on each participant using a Topcon Medical Systems (Oakland, California, USA) TRC-NW300 non-mydriatic retinal camera. One photo of each eye was taken. If an image was blurry it was repeated. Fundus photos were saved, and an ophthalmologist later interpreted these images.

### Laboratory procedures

Spot, mid-stream, clean catch urine was collected in a sterile urine cup from each participant. At least 10 mL of urine were collected in bottles labeled with the participant’s code number. The sample was then analyzed using urine strips manufactured by Acon Laboratory, Inc. (USA). The strip was read for ketones after 40 seconds, for proteins and nitrate after 60 seconds, and for leukocytes after 120 seconds.

Capillary blood was collected from a fingerpick to measure HbA1c and blood glucose with a OneTouch (Chesterbrook, Pennsylvania, USA) glucometer. The normal range for random blood glucose was 3–11 mmol/L and 3–7 mmol/L for fasting blood glucose. The HbA1c level was separated into the following classifications, using identical classifications as other studies done on diabetic children and adolescents in Tanzania [6,7]:

- HbA1c ≤ 7.5% = good glycemic control
- HbA1c 7.5–10% = moderate glycemic control
- HbA1c 10–12.5% = poor glycemic control
- HbA1c ≥ 12.5% = very poor glycemic control

Venous blood was drawn aseptically from all participants to measure serum creatinine. An estimated glomerular filtration rate (eGFR) was then calculated using the Schwartz equation and classified into stages as recommended by international guidelines [14].

### Sample size determination

The Leslie Kish formula was used to calculate sample size, using a prevalence of 29.3% for nephropathy, 22.7% for retinopathy [6], and 2.1% for neuropathy [15].

{\rm{n}} = {{\rm{Z}}^2}{\rm{p}}\left( {\left( {1 - {\rm{p}}} \right)/{{\rm{d}}^2}} \right)

n = sample size

Z = Z-score for 95% confidence interval (1.96)

p = proportion of nephropathy (0.293), proportion of retinopathy (0.22), proportion of neuropathy (0.021).

d = tolerable error (7.5% = 0.075)

The results of this formula yielded necessary sample sizes of 140 for nephropathy, 117 for retinopathy, and 14 for neuropathy. Consequently, we needed to enroll at least 140 total participants to ensure sufficient power.

### Statistical analysis

Data was entered into Microsoft Excel (Microsoft, Redmond, Washington, USA) and then exported to STATA version 13 (College Station, Texas, USA) for analysis. Continuous variables were summarized with medians and interquartile ranges, Categorical variables were summarized using frequency and proportions.

To determine the factors associated with the development of microvascular complications, univariate and multivariate logistic regression models were performed. Statistically significant variables in the univariate analysis were included in a multivariate model. Odds ratio with 95% confidence intervals were reported. A p-value of <0.05 was considered statistically significant.

### Operational definitions

We used the following operational definitions for this study, in accordance with International Society for Pediatric and Adolescent Diabetes (ISPAD) guidelines [16].

**Diabetic nephropathy**: presence of protein in urine measured by urine dipstick or eGFR less than 60 mL/min/1.73 m^2^.

**Diabetic peripheral neuropathy**: positive monofilament test with sensations of a heat or pain on the sole of the foot and upper limb.

**Diabetic autonomic neuropathy**: a decrease in blood pressure of systolic BP more 20mmgHg or diastolic more 10mmHg after three minutes of moving from a supine (sitting) to standing position along with constipation or diarrhea.

**Diabetic retinopathy**: the presence of exudates, hemorrhages, or new vessels at the disc or elsewhere in the fundus or fibrovascular membrane, reviewed by an ophthalmologist.

### Ethics approval and consent to participate

Ethical clearance was granted by Catholic University of Health and Allied Sciences, Bugando Medical Centre research ethics committee (research clearance certificate: CREC/217/2017), and Weill Cornell Medical College IRB (Protocol 1001010827). Permission to conduct the study was also obtained from Sekou Toure Regional Hospital and Sengerema Hospital. Participants were enrolled only after obtaining informed consent from a parent or guardian (or from the participant themselves if they were over the age of 18). Additionally, assent was obtained from children aged 7 to 17. All study procedures including informed consent were conducted in Kiswahili by a native speaker.

## Results

### Study enrollment

A total of 170 diabetic children and adolescents with DM attending the designated health facilities were screened for entry into the study. Of these, 155 met the eligibility criteria and were enrolled. Of the 15 excluded, six had parents/guardians who did not consent, six had parents/guardians who were not present, and three had nitrites or leukocytes present in their urine.

### Demographic and clinical data

Of 155 participants, 78 (50.3%) were male. The median age was 17 years [IQR 14,15,16,17]. Eighty-one (52.3%) of the participants resided in a rural area. Fifty-four (34.8%) had been diagnosed with diabetes for less than two years, 64 (41.2%) from two to five years, and 37 (23.9%) greater than five years. Further demographic/clinical data can be found in Table 1.

**Table 1:** Demographic and Clinical Data (n = 155).

Variable	Number (Percentage)

Median Age (IQR)	17 (14–17)
Age Range	5–19
Female	77 (49.7)
**Education of caregiver**	
None	35 (22.6)
Primary	76 (49.0)
Secondary	31 (20.0)
University	13 (8.4)
**Residence**	
Urban	74 (47.7)
Rural	81 (52.3)
**Duration of DM**	
0–2 years	54 (34.8)
2–5 years	64 (41.2)
>5 years	37 (23.9)
**Lifetime hospital admissions**	
None	33 (21.3)
One	52 (33.6)
Two	32 (20.7)
Three	19 (12.3)
More than three	19 (12.3)
**Prior blood transfusion**	12 (7.7)
**Sickle cell disease present**	3 (1.9)
**Had previous kidney screening**	36 (23.2)
**Had previous eye exam**	41 (26.5)
**Problems with vision**	
None	97 (62.6)
Blurry vision only	26 (16.8)
Floaters only	14 (9.0)
Blurry vision and floaters	18 (11.6)
**BMI**	
Underweight	20 (13.0)
Normal	101 (65.0)
Overweight	17 (11.0)
Obese	17 (11.0)
**Positive monofilament test**	29 (18.7)
**Fasting blood glucose**	
Normal	34 (21.9)
Hyperglycemia	113 (72.9)
Hypoglycemia	8 (5.2)
**Random blood glucose**	
Normal	28 (18.1)
Hyperglycemia	123 (79.3)
Hypoglycemia	4 (2.6)
**Urine dip stick results**	
**Ketones**	
Positive	27 (17.0)
**Protein**	
None	108 (69.7)
+1	43 (27.7)
+2	4 (2.6)
**Sugar**	
None	47 (30.3)
+1	41 (26.5)
+2	40 (25.8)
+3	27 (17.4)
**Glycemic Control**	
Good (<7.5%)	0 (0.0%)
Moderate (7.5–10%)	48 (31.0)
Poor (10–12.5%)	38 (24.5)
Very poor (>12.5%)	69 (44.5)
**eGFR stage (ml/min/1.73 m^2^)**	
Stage 1 (≥ 90)	124 (80.0)
Stage 2 (60–89)	18 (11.6)
Stage 3a (45–59)	9 (5.8)
Stage 3b (30–44)	4 (2.6)

### Microvascular complication prevalence

Among the 155 study participants, 65 (41.9%) met the definition for at least one microvascular complication. Fifty-one (32.9%) had diabetic nephropathy, 16 (10.3%) had diabetic retinopathy, and 21 (13.6%) had diabetic neuropathy. No micro-aneurysms were detected. Among the 21 participants with diabetic neuropathy, three (1.9%) had both peripheral and autonomic neuropathy. The prevalence of these microvascular complications is specified in Table 2.

**Table 2:** Prevalence of microvascular complications (n = 155).

Microvascular complication	Number (%)

Nephropathy	51 (32.9)
Retinopathy	16 (10.3)
Neuropathy	21 (13.6)
Autonomic neuropathy	16 (10.3)
Peripheral neuropathy	8 (5.2)
Both autonomic and peripheral neuropathy	3 (1.9)
Any complication	65 (41.9)
More than one complication	20 (12.9)

### Overlap of microvascular complications

**Figure 1:**
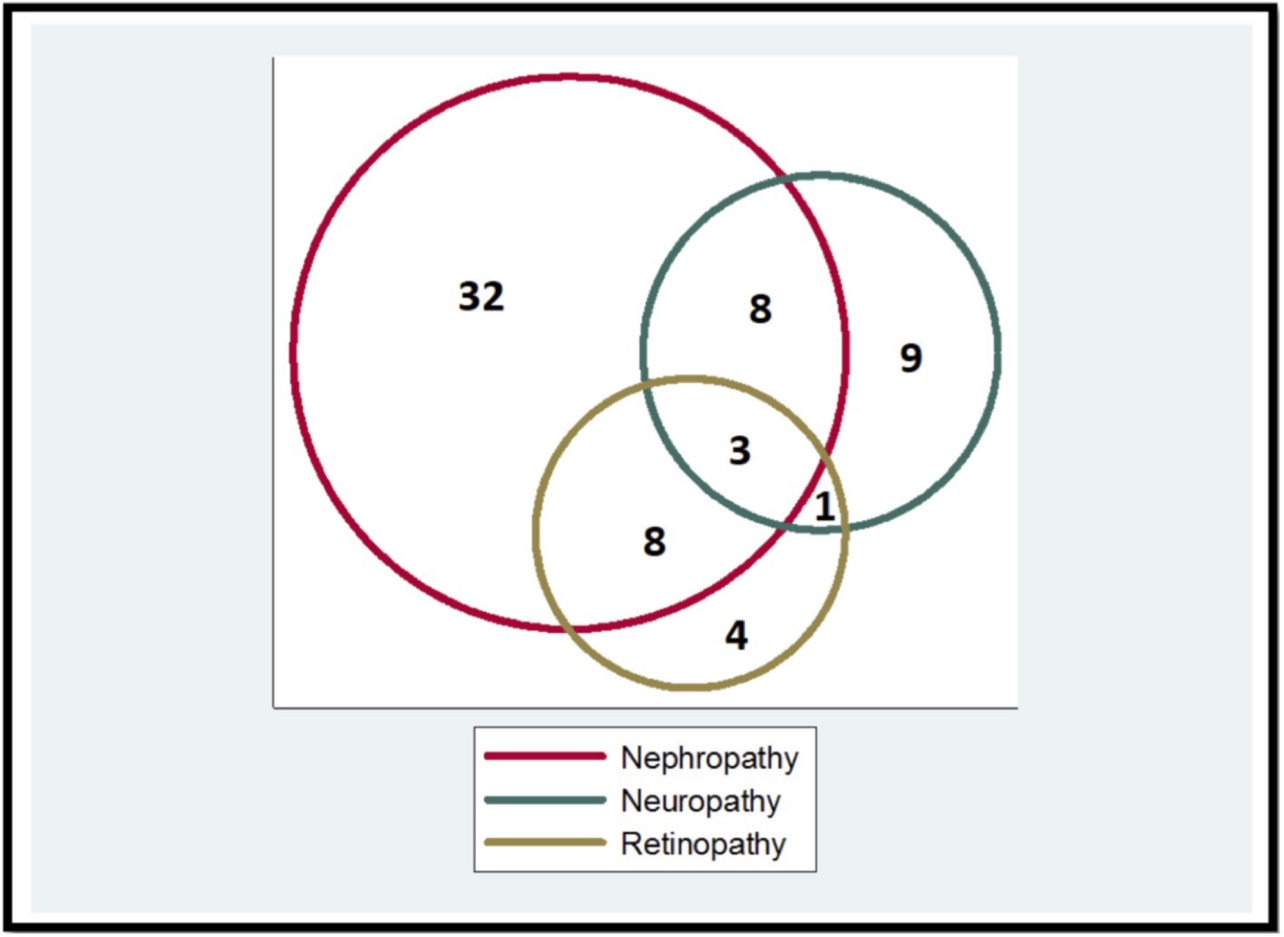
Overlap of microvascular complications (n = 65).

Of the 65 participants with any microvascular complication, 32 (49.2%) had only diabetic nephropathy, nine (13.8%) had only neuropathy and four (6.2%) had only diabetic retinopathy. Eleven (16.9%) had both nephropathy and neuropathy, 11 (16.9%) had both nephropathy and retinopathy, four (6.2%) had both neuropathy and retinopathy, and three (4.6%) had all three microvascular complications. This is summarized in Figure 1.

### Factors associated with microvascular complications

In our univariate logistic regression analysis, the factors associated with having a microvascular complication were age between 13 and 19 years (OR 11.5; 95% CI 2.5–50.5; p-value = 0.001), duration of DM greater than five years (OR 2.5; 95% CI 1.1–6.1; p-value = 0.03), and very poor glycemic control (OR 3.1; 95% CI 1.4–6.9; p-value = 0.004). In our multivariate logistic regression analysis, the factors independently associated with any microvascular complications were age between 13 and 19 years (OR 11.1; 95% CI 2.3–52.0; p-value = 0.002) and very poor glycemic control (OR 3.5; 95% CI 1.5–8.1; p-value = 0.003). These findings are summarized in Table 3.

**Table 3:** Factors associated with microvascular complications (n = 155).

Variable	Microvascular complication		Univariate analysis		Multivariate analysis	

	Yes n(%)	No n(%)	OR [95% CI]	p-value	OR [95% CI]	p-value

**Age Group**						
5–12 years	2 (7.7)	24 (92.3)	1.0	–	–	–
13–19 years	63 (48.9)	66 (51.2)	11.5 [2.5–50.5]	**0.001**	11.1 [2.3–52.0]	**0.002**
**Sex**						
Female	32 (41.6)	45 (58.4)	1.0	–	–	–
Male	33 (42.3)	45 (57.7)	0.9 [0.5–1.8]	0.925	–	–
**Education of caregiver**						
None	16 (45.7)	19 (54.3)	1.0	–	–	–
Primary	36 (47.4)	40 (52.6)	1.1 [0.4–2.4]	0.871	–	–
Secondary	10 (32.3)	21 (67.8)	0.6 [0.2–1.5]	0.266	–	–
University	3 (23.1)	10 (76.9)	0.4 [0.1–1.5]	0.163	–	–
**Residence**						
Urban	31 (41.9)	43 (58.1)	1.0	–	–	–
Rural	34 (42.0)	47 (58.0)	0.5 [0.5–1.9]	0.992	–	–
**Duration of diabetes**						
0–2 years	13 (24.1)	41 (75.9)	1.0	–	–	–
2–5 years	14 (21.9)	50 (78.1)	1.6 [0.7–3.6]	0.173	1.2 [0.5–3.8]	0.680
>5 years	13 (35.1)	24 (64.9)	2.5 [1.1–6.1]	**0.033**	2.7 [0.6–4.5]	0.292
**Number of lifetime hospital admissions**						
None	17 (51.5)	16 (48.5)	1.0	–	–	–
One	19 (36.5)	33 (63.5)	0.5 [0.2–1.3]	0.22	–	–
Two	13 (40.6)	8 (59.5)	0.6 [0.2–1.7]	0.38	–	–
Three	8 (42.1)	11 (57.9)	0.7 [0.2–2.1]	0.51	–	–
More than three	8 (42.1)	11 (57.9)	0.7 [0.2–2.1]	0.51	–	–
**Had blood transfusion?**						
Yes	7 (58.3)	5 (41.7)	2.0 [0.6–6.8]	0.239	–	–
No	58 (40.6)	85 (59.4)	1.0	–	–	–
**Sickle Cell Disease (SCD)**						
Yes	1 (33.3)	2 (66.7)	0.7 [0.1–7.7]	0.762	–	–
No	64 (42.1)	88 (57.9)	1.0	–	–	–
**Fasting blood glucose**						
Normal	19 (55.9)	15 (44.1)	1.0	–	–	–
Hyperglycemia	43 (38.1)	70 (62.0)	0.5 [0.2–1.1]	0.068	–	–
Hypoglycemia	3 (37.5)	5 (62.5)	0.5 [0.1–2.3]	0.355	–	–
**Random blood glucose**						
Normal	15 (53.6)	13 (46.4)	1.0	–	–	–
Hyperglycemia	49 (39.8)	74 (60.2)	0.5 [0.3–1.3]	0.187	–	–
Hypoglycemia	1 (25.0)	3 (75.0)	0.2 [0.03–3.1]	0.307	–	–
**Glycemic Control (HbA1c level)**						
Moderate (7.5–10%)	14 (29.2)	34 (70.8)	1.0	–	–	–
Poor (10–12.5%)	12 (31.6)	26 (68.4)	1.1 [0.4–2.8]	0.809	1.1 [0.4–2.9]	0.822
Very poor (>12.5%) (>12.5%)	39 (56.5)	30 (43.5)	3.1 [1.4–6.9]	**0.004**	3.5 [1.5–8.1]	**0.003**

## Discussion

Nearly one-third of the participants in this study showed evidence of diabetic nephropathy, a very high prevalence compared to other countries in the region. Nephropathy rates in similar studies on diabetic children and adolescents in Africa ranged from 0–29%, all lower than this study [6,15,17,18]. Even some research in adult populations in Africa has found lower nephropathy rates than here [19,20], though age is a significant risk factor for development of microvascular complications [21,22]. One possible explanation for the elevated rate of nephropathy is that it stems from the high levels of kidney damage already existing in children in SSA [11]. In the Lake Zone region of Western Tanzania in particular, schistosomiasis may be an important infectious contributor to nephropathy [23].

While the prevalence of neuropathy and retinopathy found here were not as high as that of nephropathy, they were still higher than expected. The prevalence of neuropathy and retinopathy in African children and adolescents with DM ranged from 2.1–6.3% and 1.8–22% in other studies [6,15,17,18], compared to 13.6% and 10.3% here. Some research in children and adolescents with DM done in the USA and Australia found rates of microvascular complication that were similar or even higher than those found here, but these studies used more sensitive diagnostic tests [16,24]. Most research from Western countries showed significantly lower microvascular complication rates, such as one study done in in Ankara, Turkey that found a 16.1% rate of microalbuminuria, 0.6% rate of neuropathy, and no cases of retinopathy [25].

Factors significantly associated with the development of microvascular complications in our study – older age, duration of diabetes, and poor glycemic control –were similar to those described in both low and high income populations [21,22,26].

Poor glycemic control is a major contributor to the development of microvascular complications and is thus a major threat to the lives of children and adolescents with DM throughout Africa. In our study, over two-thirds of participants had poor glycemic control (HbA1C > 10%). Other studies in children and adolescents from Rwanda, Egypt, and Ethiopia demonstrated equally high rates of poor glycemic control [15,17,18]. Poor glycemic control was the strongest modifiable risk factor for microvascular complications in our study population. The landmark Diabetes Control and Complications Trial (DCCT) demonstrated that a drop in HbA1c of 2% reduced the risk of retinopathy by 63%, nephropathy by 54%, and neuropathy by 60% [21]. Therefore, improving glycemic control should be the first priority for preventing microvascular complications and promoting health in children with DM in Africa.

There are limitations to our study. This was a cross-sectional study and therefore it was only hypothesis generating. Furthermore, the nephropathy diagnostic method used here, a random urine dip-stick, is potentially less sensitive than the recommended method of albumin to creatinine measurement of first morning urine samples or kidney biopsy. However, a similar study done on adults with DM at the same hospital also found remarkably high levels of kidney disease, suggesting that the high rate found here is feasible [27]. The diagnostic method used in this research is also the typical diagnostic method at our hospital and at many other hospitals in similar settings. Additionally, we did not test for proprioception or vibration when diagnosing peripheral neuropathy.

## Conclusions

Microvascular complications are extremely common in children and adolescents with DM in Tanzania, higher even than surrounding regions. Nearly one-third of the participants in our study demonstrated evidence of diabetic nephropathy. Besides age, the strongest independent predictor of microvascular complications was HbA1C level, and two-thirds of the participants met criteria for poor glycemic control. Health systems for DM care must be strengthened in Africa to ensure adequate resources for early diagnosis and management of microvascular complications. In addition, interventions are urgently needed to improve the long-term health of children and adolescents with DM in Africa.

## Abbreviations

Diabetes mellitus – DM

Estimated glomerular filtration rate – eGFR

Sub-Saharan Africa – SSA
